# Crystal structure of *catena*-poly[[chlorido­(4,4′-dimethyl-2,2′-bi­pyridine-κ^2^ *N*,*N*′)copper(II)]-μ-chlorido]

**DOI:** 10.1107/S2056989015008944

**Published:** 2015-05-13

**Authors:** Rafaela Nita, Jeffrey R. Deschamps, Scott A. Trammell, D. Andrew Knight

**Affiliations:** a Chemistry Department, Florida Institute of Technology, 150 West University Boulevard, Melbourne, FL 32901, USA; b Naval Research Laboratory, 4555 Overlook Ave, Washington, DC 20375, USA

**Keywords:** crystal structure, copper bi­pyridine complex, dehydration, hydrogen bonding

## Abstract

The title compound, [CuCl_2_(C_12_H_12_N_2_)]_*n*_, was obtained *via* a DMSO-mediated dehydration of Cu(4,4′-dimethyl-2,2′-bi­pyridine)­copper(II)·0.25H_2_O. The central Cu^II^ atom is coordinated in a distorted trigonal–bipyramidal geometry by two N atoms of a chelating 4,4′-dimethyl-2,2′-bi­pyridine ligand [average Cu—N = 2.03 (3) Å] and three Cl atoms, one terminal with a short Cu—Cl bond of 2.2506 (10) Å, and two symmetry-equivalent and bridging bonds. The bridging Cl atom links the Cu^II^ ions into chains parallel to [001] *via* one medium and one long Cu—Cl bond [2.3320 (10) and 2.5623 (9) Å]. The structure displays both inter- and intra­molecular C—H⋯Cl hydrogen bonding.

## Abstract 2

The structure of a previously unknown form of di­chlorido­(4,4′-dimethyl-2,2′-bi­pyridine)­copper(II) was obtained *via* a DMSO-mediated dehydration of Cu(4,4′-dimethyl-2,2′-bi­pyridine)­copper(II)·0.25H_2_O. The crystal structure reveals chloride-bridged copper(II) chains connected *via* inter­molecular C—H⋯Cl hydrogen bonds.

## Chemical context   

Bi­pyridine complexes of copper(II), [(2,2′-bipy)Cu*X*_2_] (*X* = Cl, Br) have been used in a number of important applications in recent years, most notably in the areas of catalysis for organic synthesis (Ricardo *et al.*, 2008 ▸; Csonka *et al.*, 2008 ▸; Thorpe *et al.*, 2012 ▸), DNA cleavage (Jaividhya *et al.*, 2012 ▸), degradation of pesticides (Knight *et al.*, 2014 ▸) and water oxidation (Barnett *et al.*, 2012 ▸). Such complexes are characterized by an extensive number of metal coordination geometries including square-planar/tetra­hedral, square-pyramidal/trigonal–bipyramidal and distorted octa­hedral. The associated halide ligands (chloride, bromide) can adopt terminal or bridging bonding modes leading to monomeric, dimeric or polymeric chain structures which can influence complex solubility in organic solvents and consequently their possible application in homogeneous catalysis. A third factor which influences the structural forms of these complexes is the nature of the solvent, with strongly coordinating ligands forming solvent adducts. For example, the reaction of dimethyl-2,2′-bi­pyridine with Cu^I^ and/or Cu^II^ in DMSO or water led to the isolation of 10 different crystalline materials, suggesting that a large number of structural motifs are possible including five-coordinate monomers, distorted tetra­hedral monomers, stacked planar monomers, stacked planar bibridged dimers and and five-coordinate bibridged dimers (Willett *et al.*, 2001 ▸). A large number of ring-substituted 2,2′-bi­pyridine complexes have also been prepared and characterized including di­chlorido­(4,4′-dimethyl-2,2′-bi­pyridine) copper(II) hemihydate. In this paper we describe the synthesis and structural characterization of a previously unknown form of di­chlorido­(4,4′-dimethyl-2,2′-bi­pyridine)­copper(II) *via* a DMSO-mediated dehydration of Cu(4,4′-dimethyl-2,2′-bi­pyri­dine)Cl_2_·0.25H_2_O. The crystal structure reveals single chlorido-bridged copper(II) chains with a distorted trigonal–bipyramidal geometry of the metal cations. We conclude that the presence of the 4,4′-dimethyl substituents does not prevent the formation of a catenated structure, which was previously suggested as an explanation for the dimeric arrangement in Cu(4,4′-dimethyl-2,2′-bi­pyridine)Cl_2_·0.5H_2_O (González *et al.*, 1993 ▸).
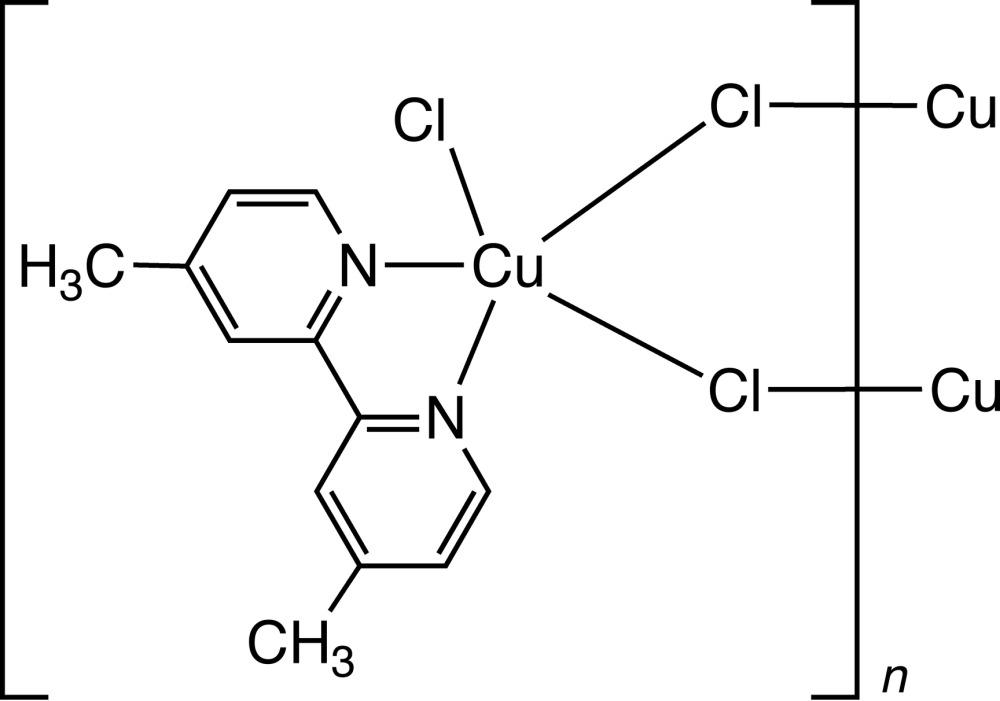


## Structural commentary   

**Figure 1:**
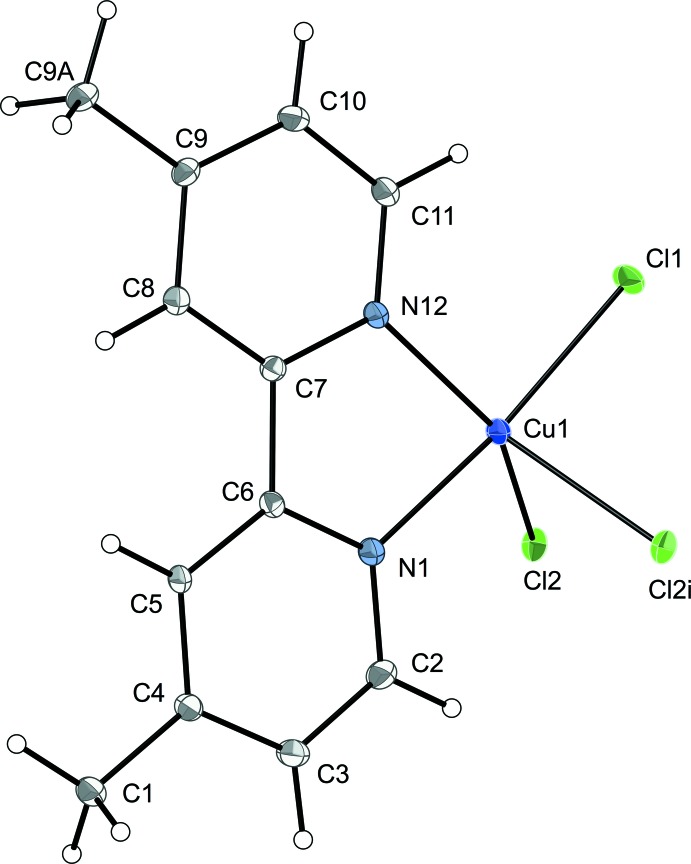
*ORTEP*-style view of compound (**1**), showing the atom-numbering scheme. Displacement ellipsoids are drawn at the 50% probability level. [Symmetry code: (i) *x* − 1, −*y* + 2, *z* − 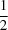.]

In the title complex (**1**), Fig. 1 ▸, the central Cu^II^ atom is coord­inated by the two nitro­gen atoms, N1 and N12 of the chelating 2,2′-bi­pyridine subunit and three chlorine atoms, one terminal (Cl1) with a short Cu—Cl bond, and two bridging chlorine atoms (Cl2), which are symmetry equivalent. The bridging chlorine ligand links Cu atoms into chains *via* one medium and one long Cu—Cl bond [2.3320 (10) and 2.5623 (9) Å]. The geometry around the Cu ion is best described as a distorted trigonal bipyramid with the coordin­ation polyhedron defined by the two N atoms and three Cl atoms, one of which links the monomeric subunits into a chain, which contrasts with the four-coordinate square-planar geometry found in Cu(2,2′-bi­pyridine)Cl_2_ (Wang *et al.*, 2004 ▸; Garland *et al.*, 1988 ▸). The two axial sites are occupied by N1 and Cl1 [N1—Cu1—Cl1 = 172.93 (10)°] and the basal plane contains the N12 atom, the Cl2 atom and the bridging Cl2 atom. The terminal Cu1—Cl1 and medium-length bridging Cu1—Cl2 bond lengths in (**1**) are 2.2506 (10) and 2.3320 (10) Å which are comparable to those found in the related structure Cu(2,2′-bi­pyridine)Cl_2_ [2.254 (4) Å; Wang *et al.*, 2004 ▸] and its polymorph [2.291 (3) Å; Hernández-Molina *et al.*, 1999 ▸], and in di­chlorido­(4,4′-dimeth­yl)-2,2′-bi­pyridine)­copper(II) hemihydrate [2.255 (2) and 2.274 (2) Å, respectively; González *et al.*, 1993 ▸]. However, the longer bridging Cu—Cl bond has a length of 2.5623 (9) Å which is shorter than those found in the above comparison structures [3.047 (3), 2.674 (3) and 2.754 (2) Å]. The Cu—N1 and Cu—N12 bond lengths in (**1**) are 2.009 (3) and 2.047 (3) Å, similar to those found in the above structures [2.024 (6), 2.037 (8), and 2.001 (3) and 2.035 (4) Å, respectively]. These comparisons indicate that neither hydration nor 4,4′-dialkyl substitution significantly affects either the terminal Cu—Cl or Cu—N bond lengths. The bi­pyridine ring presents a bite angle of 79.25 (12)° to Cu, similar to that found in the above-mentioned structures, 80.5 (3), 79.6 (3) and 80.2 (1)° respectively, and forming a virtually planar five-membered ring. The C—C and C—N bond lengths and angles are within expected limits.

## Supra­molecular features   

**Figure 2:**
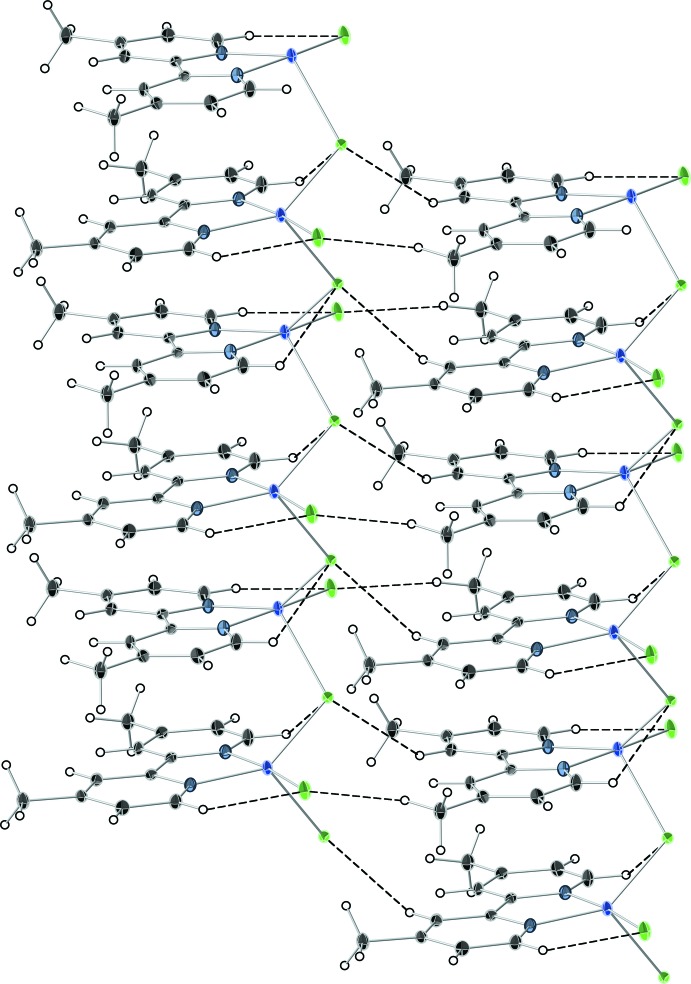
Selected portion of the crystal packing diagram of compound (**1**), showing inter­chain C—H⋯Cl hydrogen bonding (see Table 1 ▸ for details).

The crystal structure of (**1**) can best be described as a linear polymer consisting of monomeric units with chains extending parallel to [001]. The chains are connected *via* weak C—H⋯Cl hydrogen bonds (Table 1 ▸ and Fig. 2 ▸). Adjacent copper atoms are bridged *via* single chlorine atoms [Cu1—Cl2^i^ = 2.5623 (9) Å; (i) = *x*, −*y* + 2, *z* − 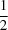). This contrasts with the structure found in Cu(2,2′-bi­pyridine)Cl_2_ in which two chlorine atoms link the monomeric substructures into a catenated complex. In (**1**) an intra­molecular C—H⋯Cl hydrogen bond is also observed (Table 1 ▸).

## Database survey   

A large number of unsubstituted and substituted bi­pyridine copper complexes with halide ligands can be found in the Cambridge Structural Database (CSD, Version 5.35; Groom & Allen, 2015 ▸). These structures have four-, five, and six-coord­ination. The related structure di­chlorido­(4,4′-dimeth­yl)-2,2′-bi­pyridine)­copper(II) hemihydrate (González *et al.*, 1993 ▸) crystallizes with a dimeric arrangement of subunits. The unsubstituted complex Cu(2,2′-bi­pyridine)Cl_2_ has been found to form both simple monomeric (Kostakis *et al.*, 2006 ▸) and chain structures (Hernández-Molina *et al.*, 1999 ▸; Wang *et al.*, 2004 ▸), the latter bearing similarities to the structure of (**1**).

## Synthesis and crystallization   

Solvents and reagents were obtained and purified as follows: DMSO (Aldrich), dried over 4 Å mol­ecular sieves, CuCl_2_·2H_2_O, 4,4′-dimethyl-2,2′-bi­pyridine (Sigma–Aldrich) used as received. Cu(4,4′-dimethyl-2,2′-bi­pyridine)Cl_2_·0.25 H_2_O was prepared according to the literature procedure (Moore *et al.*, 2012 ▸). Cu(4,4′-dimethyl-2,2′-bi­pyridine)Cl_2_·0.25 H_2_O (0.4091 g, 1.266 mmol) was dissolved in anhydrous DMSO (500 ml) and stored at 277 K for 30 months (shorter periods of time, *e.g.* 7 days, did not result in dehydration). The DMSO was then removed under a stream of N_2_ and the resulting solid was further dried *in vacuo* at 313 K to give (**1**) as a green powder (0.386 g, 1.21 mmol, 96% yield). A portion of (**1**) was dissolved in DMSO and concentrated under a stream of N_2_ (flow rate = 12 l/min) over 7 days in an open vial to give green plates. Analysis calculated for CuC_12_H_12_N_2_Cl_2_: C, 45.23; H, 3.80; N, 8.79. Found: C, 44.69; H, 3.66; N, 8.20.

## Refinement   

Crystal data, data collection and structure refinement details are summarized in Table 2 ▸. The H atoms were included in calculated positions and refined as riding: C—H = 0.95–0.98 Å with *U*_iso_(H) = 1.5*U*_eq_(C) for methyl H atoms and 1.2*U*_eq_(C) for other H atoms.

## Supplementary Material

Crystal structure: contains datablock(s) I. DOI: 10.1107/S2056989015008944/zl2617sup1.cif

Structure factors: contains datablock(s) I. DOI: 10.1107/S2056989015008944/zl2617Isup2.hkl

CCDC reference: 1063931

Additional supporting information:  crystallographic information; 3D view; checkCIF report

## Figures and Tables

**Table 1:** Hydrogen-bond geometry (, )

*D*H*A*	*D*H	H*A*	*D* *A*	*D*H*A*
C11H11*A*Cl1	0.95	2.61	3.211(4)	122
C8H8*A*Cl2^i^	0.95	2.88	3.666(4)	140
C10H10*A*Cl1^ii^	0.95	2.88	3.733(4)	149

Symmetry codes: (i) 
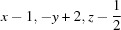
; (ii) 
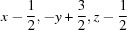
.

**Table 2:** Experimental details

Crystal data	
Chemical formula	[CuCl_2_(C_12_H_12_N_2_)]
*M*_r_	318.68
Crystal system, space group	Monoclinic, *C* *c*
Temperature (K)	150
*a*, *b*, *c* ()	9.1101(6), 20.0087(12), 7.1231(4)
()	110.491(2)
*V* (^3^)	1216.25(13)
*Z*	4
Radiation type	Mo *K*
(mm^1^)	2.21
Crystal size (mm)	0.27 0.12 0.07

Data collection	
Diffractometer	Bruker APEXII CCD
Absorption correction	Multi-scan (*SADABS*; Bruker, 2002 ▸)
*T*_min_, *T*_max_	0.646, 0.746
No. of measured, independent and observed [*I* > 2(*I*)] reflections	7099, 2945, 2829
*R*_int_	0.049
(sin /)_max_ (^1^)	0.685

Refinement	
*R*[*F* ^2^ > 2(*F* ^2^)], *wR*(*F* ^2^), *S*	0.030, 0.072, 1.05
No. of reflections	2945
No. of parameters	156
No. of restraints	2
H-atom treatment	H-atom parameters constrained
_max_, _min_ (e ^3^)	0.56, 0.48
Absolute structure	Classical Flack method preferred over Parsons because s.u. lower (Flack, 1983 ▸)
Absolute structure parameter	0.011(15)

Computer programs: *SMART*, *SAINT* and *XPREP* (Bruker, 2002 ▸), *SHELXS97* and *SHELXTL* (Sheldrick, 2008 ▸) and *SHELXL2014* (Sheldrick, 2015 ▸).

## supplementary crystallographic information

### Crystal data

**Table:**

[CuCl_2_(C_12_H_12_N_2_)]	*F*(000) = 644
*M**_r_* = 318.68	*D*_x_ = 1.740 Mg m^−^^3^
Monoclinic, *C**c*	Mo *K*α radiation, λ = 0.71073 Å
*a* = 9.1101 (6) Å	Cell parameters from 4788 reflections
*b* = 20.0087 (12) Å	θ = 2.6–29.1°
*c* = 7.1231 (4) Å	µ = 2.21 mm^−^^1^
β = 110.491 (2)°	*T* = 150 K
*V* = 1216.25 (13) Å^3^	Plate, green
*Z* = 4	0.27 × 0.12 × 0.07 mm

### Data collection

**Table:**

Bruker APEXII CCD diffractometer	2945 independent reflections
Radiation source: sealed tube	2829 reflections with *I* > 2σ(*I*)
Graphite monochromator	*R*_int_ = 0.049
ω scans	θ_max_ = 29.1°, θ_min_ = 2.0°
Absorption correction: multi-scan (*SADABS*; Bruker, 2002)	*h* = −12→12
*T*_min_ = 0.646, *T*_max_ = 0.746	*k* = −27→27
7099 measured reflections	*l* = −9→9

### Refinement

**Table:**

Refinement on *F*^2^	Secondary atom site location: difference Fourier map
Least-squares matrix: full	Hydrogen site location: inferred from neighbouring sites
*R*[*F*^2^ > 2σ(*F*^2^)] = 0.030	H-atom parameters constrained
*wR*(*F*^2^) = 0.072	*w* = 1/[σ^2^(*F*_o_^2^) + (0.0425*P*)^2^] where *P* = (*F*_o_^2^ + 2*F*_c_^2^)/3
*S* = 1.05	(Δ/σ)_max_ = 0.001
2945 reflections	Δρ_max_ = 0.56 e Å^−^^3^
156 parameters	Δρ_min_ = −0.48 e Å^−^^3^
2 restraints	Absolute structure: Classical Flack method preferred over Parsons because s.u. lower (Flack, 1983).
Primary atom site location: structure-invariant direct methods	Absolute structure parameter: 0.011 (15)

### Special details

**Table:**

Geometry. All e.s.d.'s (except the e.s.d. in the dihedral angle between two l.s. planes) are estimated using the full covariance matrix. The cell e.s.d.'s are taken into account individually in the estimation of e.s.d.'s in distances, angles and torsion angles; correlations between e.s.d.'s in cell parameters are only used when they are defined by crystal symmetry. An approximate (isotropic) treatment of cell e.s.d.'s is used for estimating e.s.d.'s involving l.s. planes.

### Fractional atomic coordinates and isotropic or equivalent isotropic displacement parameters (Å^2^)

**Table:**

	*x*	*y*	*z*	*U*_iso_*/*U*_eq_	
Cu1	0.99673 (5)	0.95231 (2)	0.74601 (5)	0.01585 (12)	
Cl1	1.12820 (11)	0.85482 (5)	0.80882 (16)	0.0243 (2)	
Cl2	1.15771 (10)	1.00184 (5)	1.04309 (13)	0.01687 (18)	
N1	0.8565 (4)	1.03282 (16)	0.6685 (5)	0.0166 (6)	
C2	0.9078 (5)	1.0962 (2)	0.6952 (6)	0.0217 (8)	
H2A	1.0170	1.1044	0.7567	0.026*	
C3	0.8073 (5)	1.14974 (19)	0.6364 (6)	0.0213 (8)	
H3A	0.8479	1.1940	0.6576	0.026*	
C4	0.6467 (5)	1.13963 (18)	0.5460 (6)	0.0164 (7)	
C4A	0.5357 (5)	1.19727 (19)	0.4831 (7)	0.0219 (8)	
H4AA	0.4287	1.1817	0.4592	0.033*	
H4AB	0.5414	1.2168	0.3597	0.033*	
H4AC	0.5642	1.2311	0.5892	0.033*	
C5	0.5941 (5)	1.07339 (18)	0.5156 (6)	0.0155 (6)	
H5A	0.4856	1.0641	0.4530	0.019*	
C6	0.7009 (4)	1.02135 (18)	0.5771 (5)	0.0136 (6)	
C7	0.6593 (4)	0.94980 (17)	0.5520 (5)	0.0137 (7)	
C8	0.5058 (4)	0.9266 (2)	0.4740 (6)	0.0167 (7)	
H8A	0.4208	0.9573	0.4344	0.020*	
C9	0.4773 (5)	0.85789 (19)	0.4542 (6)	0.0162 (7)	
C9A	0.3132 (5)	0.8319 (2)	0.3748 (7)	0.0225 (8)	
H9AA	0.2582	0.8514	0.2425	0.034*	
H9AB	0.2588	0.8441	0.4666	0.034*	
H9AC	0.3151	0.7832	0.3630	0.034*	
C10	0.6064 (5)	0.81541 (19)	0.5120 (6)	0.0192 (7)	
H10A	0.5919	0.7684	0.4982	0.023*	
C11	0.7558 (5)	0.84178 (19)	0.5896 (6)	0.0191 (7)	
H11A	0.8425	0.8120	0.6290	0.023*	
N12	0.7834 (3)	0.90766 (15)	0.6114 (5)	0.0153 (6)	

### Atomic displacement parameters (Å^2^)

**Table:**

	*U*^11^	*U*^22^	*U*^33^	*U*^12^	*U*^13^	*U*^23^
Cu1	0.01070 (19)	0.0139 (2)	0.0205 (2)	0.00155 (17)	0.00239 (16)	0.00001 (18)
Cl1	0.0162 (4)	0.0162 (4)	0.0347 (5)	0.0053 (3)	0.0016 (4)	0.0024 (4)
Cl2	0.0135 (4)	0.0235 (4)	0.0134 (4)	−0.0012 (3)	0.0044 (3)	−0.0025 (3)
N1	0.0131 (15)	0.0153 (14)	0.0203 (15)	0.0005 (12)	0.0046 (13)	−0.0014 (12)
C2	0.0144 (18)	0.0188 (18)	0.029 (2)	−0.0023 (14)	0.0042 (16)	−0.0002 (15)
C3	0.0200 (19)	0.0150 (17)	0.026 (2)	−0.0012 (14)	0.0050 (17)	−0.0016 (15)
C4	0.0171 (17)	0.0148 (17)	0.0170 (17)	0.0004 (13)	0.0057 (14)	−0.0009 (13)
C4A	0.0179 (18)	0.0161 (18)	0.030 (2)	0.0021 (15)	0.0061 (16)	−0.0006 (16)
C5	0.0107 (15)	0.0151 (16)	0.0198 (18)	0.0020 (14)	0.0043 (14)	−0.0005 (14)
C6	0.0143 (16)	0.0144 (16)	0.0133 (16)	0.0018 (13)	0.0063 (13)	0.0007 (13)
C7	0.0149 (17)	0.0130 (16)	0.0140 (17)	0.0000 (13)	0.0062 (15)	−0.0001 (12)
C8	0.0148 (19)	0.0168 (18)	0.0184 (17)	0.0005 (13)	0.0057 (15)	−0.0008 (14)
C9	0.0154 (17)	0.0172 (17)	0.0161 (17)	−0.0030 (13)	0.0057 (14)	−0.0018 (14)
C9A	0.017 (2)	0.0190 (19)	0.029 (2)	−0.0051 (15)	0.0047 (17)	−0.0032 (16)
C10	0.0202 (18)	0.0128 (16)	0.0239 (19)	0.0004 (14)	0.0069 (16)	0.0013 (14)
C11	0.0161 (18)	0.0154 (17)	0.025 (2)	0.0028 (13)	0.0062 (16)	−0.0002 (14)
N12	0.0123 (14)	0.0136 (14)	0.0191 (15)	0.0022 (12)	0.0044 (12)	0.0000 (12)

### Geometric parameters (Å, º)

**Table:**

Cu1—N1	2.009 (3)	C5—C6	1.387 (5)
Cu1—N12	2.047 (3)	C5—H5A	0.9500
Cu1—Cl1	2.2506 (10)	C6—C7	1.476 (5)
Cu1—Cl2	2.3320 (10)	C7—N12	1.354 (4)
Cu1—Cl2^i^	2.5623 (9)	C7—C8	1.391 (5)
Cl2—Cu1^ii^	2.5623 (9)	C8—C9	1.398 (5)
N1—C2	1.343 (5)	C8—H8A	0.9500
N1—C6	1.357 (5)	C9—C10	1.391 (5)
C2—C3	1.375 (6)	C9—C9A	1.494 (5)
C2—H2A	0.9500	C9A—H9AA	0.9800
C3—C4	1.392 (5)	C9A—H9AB	0.9800
C3—H3A	0.9500	C9A—H9AC	0.9800
C4—C5	1.400 (5)	C10—C11	1.382 (6)
C4—C4A	1.495 (5)	C10—H10A	0.9500
C4A—H4AA	0.9800	C11—N12	1.341 (5)
C4A—H4AB	0.9800	C11—H11A	0.9500
C4A—H4AC	0.9800		
			
N1—Cu1—N12	79.25 (12)	C6—C5—H5A	120.1
N1—Cu1—Cl1	172.93 (10)	C4—C5—H5A	120.1
N12—Cu1—Cl1	93.82 (9)	N1—C6—C5	121.6 (4)
N1—Cu1—Cl2	92.64 (10)	N1—C6—C7	113.8 (3)
N12—Cu1—Cl2	143.41 (9)	C5—C6—C7	124.6 (4)
Cl1—Cu1—Cl2	93.79 (4)	N12—C7—C8	122.0 (3)
N1—Cu1—Cl2^i^	89.55 (9)	N12—C7—C6	114.5 (3)
N12—Cu1—Cl2^i^	121.94 (9)	C8—C7—C6	123.4 (3)
Cl1—Cu1—Cl2^i^	93.01 (4)	C7—C8—C9	119.5 (4)
Cl2—Cu1—Cl2^i^	93.29 (3)	C7—C8—H8A	120.2
Cu1—Cl2—Cu1^ii^	111.20 (4)	C9—C8—H8A	120.2
C2—N1—C6	118.8 (3)	C10—C9—C8	117.6 (4)
C2—N1—Cu1	124.2 (3)	C10—C9—C9A	122.0 (4)
C6—N1—Cu1	117.0 (2)	C8—C9—C9A	120.4 (4)
N1—C2—C3	122.1 (4)	C9—C9A—H9AA	109.5
N1—C2—H2A	119.0	C9—C9A—H9AB	109.5
C3—C2—H2A	119.0	H9AA—C9A—H9AB	109.5
C2—C3—C4	120.5 (3)	C9—C9A—H9AC	109.5
C2—C3—H3A	119.7	H9AA—C9A—H9AC	109.5
C4—C3—H3A	119.7	H9AB—C9A—H9AC	109.5
C3—C4—C5	117.2 (3)	C11—C10—C9	119.8 (3)
C3—C4—C4A	121.2 (3)	C11—C10—H10A	120.1
C5—C4—C4A	121.7 (4)	C9—C10—H10A	120.1
C4—C4A—H4AA	109.5	N12—C11—C10	122.7 (3)
C4—C4A—H4AB	109.5	N12—C11—H11A	118.6
H4AA—C4A—H4AB	109.5	C10—C11—H11A	118.6
C4—C4A—H4AC	109.5	C11—N12—C7	118.3 (3)
H4AA—C4A—H4AC	109.5	C11—N12—Cu1	126.3 (3)
H4AB—C4A—H4AC	109.5	C7—N12—Cu1	115.3 (2)
C6—C5—C4	119.9 (4)		
			
C6—N1—C2—C3	1.2 (6)	N1—C6—C7—C8	175.7 (3)
Cu1—N1—C2—C3	178.8 (3)	C5—C6—C7—C8	−4.1 (6)
N1—C2—C3—C4	0.1 (6)	N12—C7—C8—C9	−0.5 (6)
C2—C3—C4—C5	−1.0 (6)	C6—C7—C8—C9	179.5 (3)
C2—C3—C4—C4A	179.3 (4)	C7—C8—C9—C10	−0.9 (6)
C3—C4—C5—C6	0.7 (5)	C7—C8—C9—C9A	178.7 (3)
C4A—C4—C5—C6	−179.7 (4)	C8—C9—C10—C11	1.3 (6)
C2—N1—C6—C5	−1.6 (5)	C9A—C9—C10—C11	−178.3 (4)
Cu1—N1—C6—C5	−179.3 (3)	C9—C10—C11—N12	−0.4 (6)
C2—N1—C6—C7	178.6 (3)	C10—C11—N12—C7	−1.0 (6)
Cu1—N1—C6—C7	0.9 (4)	C10—C11—N12—Cu1	174.3 (3)
C4—C5—C6—N1	0.6 (6)	C8—C7—N12—C11	1.4 (6)
C4—C5—C6—C7	−179.7 (3)	C6—C7—N12—C11	−178.6 (3)
N1—C6—C7—N12	−4.3 (4)	C8—C7—N12—Cu1	−174.4 (3)
C5—C6—C7—N12	176.0 (4)	C6—C7—N12—Cu1	5.6 (4)

Symmetry codes: (i) *x*, −*y*+2, *z*−1/2; (ii) *x*, −*y*+2, *z*+1/2.

### Hydrogen-bond geometry (Å, º)

**Table:**

*D*—H···*A*	*D*—H	H···*A*	*D*···*A*	*D*—H···*A*
C11—H11*A*···Cl1	0.95	2.61	3.211 (4)	122
C8—H8*A*···Cl2^iii^	0.95	2.88	3.666 (4)	140
C10—H10*A*···Cl1^iv^	0.95	2.88	3.733 (4)	149

Symmetry codes: (iii) *x*−1, −*y*+2, *z*−1/2; (iv) *x*−1/2, −*y*+3/2, *z*−1/2.
